# Investigation of high-pressure planetary ices by cryo-recovery. II. High-pressure apparatus, examples and a new high-pressure phase of MgSO_4_·5H_2_O

**DOI:** 10.1107/S1600576718003977

**Published:** 2018-04-27

**Authors:** Weiwei Wang, A. Dominic Fortes, David P. Dobson, Christopher M. Howard, John Bowles, Neil J. Hughes, Ian G. Wood

**Affiliations:** a Department of Earth Sciences, University College London, Gower Street, London WC1E 6BT, UK; b ISIS Facility, STFC Rutherford Appleton Laboratory, Harwell Science and Innovation Campus, Chilton, Oxfordshire OX11 0QX, UK

**Keywords:** planetary ices, high pressure, cryo-recovery, epsomite, magnesium sulfate pentahydrate

## Abstract

An apparatus is described for the compression of samples to ∼2 GPa at temperatures from 80 to 300 K, rapid chilling to 80 K whilst under load and subsequent recovery into liquid nitro­gen after the load is released. In this way, a variety of quenchable high-pressure phases of many materials may be preserved for examination outside the high-pressure sample environment, with the principal benefit being the ability to obtain high-resolution powder diffraction data for phase identification and structure solution. The use of this apparatus, in combination with a newly developed cold-loadable low-temperature stage for X-ray powder diffraction (the PheniX-FL), is illustrated using ice VI (a high-pressure polymorph of ordinary water ice that is thermodynamically stable only above ∼0.6 GPa) as an example. A second example using synthetic epsomite (MgSO_4_·7H_2_O) reveals that, at ∼1.6 GPa and 293 K, it undergoes incongruent melting to form MgSO_4_·5H_2_O plus brine, contributing to a long-standing debate on the nature of the high-pressure behaviour of this and similar highly hydrated materials. The crystal structure of this new high-pressure polymorph of MgSO_4_·5H_2_O has been determined at 85 K in space group *Pna*2_1_ from the X-ray powder diffraction pattern of a sample recovered into liquid nitro­gen and is found to differ from that of the known ambient-pressure phase of MgSO_4_·5H_2_O (pentahydrite, space group ), consisting of corner-sharing MgO_6_–SO_4_ ion pairs rather than infinite corner-sharing chains.

## Abstract 2

An apparatus for the compression of materials to ∼2 GPa and subsequent rapid chilling to 80 K with extraction to ambient pressure is described. The partial resolution of a long-standing problem concerning the high-pressure phase behaviour of a water-rich salt hydrate is demonstrated using the equipment.

## 1. Introduction   

In an accompanying paper (Wood *et al.*, 2018 ▸) we describe a new low-temperature stage (the PheniX-FL) for X-ray powder diffraction operating at temperatures (*T*) 40 ≤ *T* ≤ 315 K. A unique feature of this apparatus is that samples may be introduced into (and removed from) the stage at any temperature in the range 80–300 K and thus materials that are not stable at room temperature can be readily examined. Such materials fall into two classes: (i) those which are thermodynamically stable at atmospheric pressure (*P*), and (ii) those, such as high-pressure ‘planetary ices’, which are truly stable only at high *P*, but which may be recovered metastably to atmospheric pressure by quenching (for example into liquid nitro­gen). For many of these planetary ices (one example being water ice itself) the kinetics of transformation to the thermodynamically stable polymorph are very slow below ∼150 K and the materials are effectively permanently metastable below this temperature. Preparation and examination of such materials by high-pressure synthesis and quenching has the advantage that they may then be studied outside a pressure cell, removing factors such as the substantial beam attenuation and parasitic scattering from the high-pressure environment and (especially on neutron powder diffractometers with fixed detectors) geometric restrictions on the accessible data. In general, therefore, this procedure allows collection of the highest-resolution data, with good counting statistics, to be more easily accomplished, thereby facilitating, for example, the determination of unknown crystal structures or accurate measurements of physical properties. Results obtained in this way may then be used to aid interpretation of measurements made at high pressure, within the material’s stability field, or may be compared with the properties of the material as predicted by computer simulations.

This approach to the study of high-pressure phases has been used previously in combination with neutron single-crystal and powder diffraction (*e.g.* La Placa *et al.*, 1973 ▸; Klotz *et al.*, 1999 ▸, 2005 ▸; Hansen *et al.*, 2008 ▸; Millar *et al.*, 2010 ▸) and X-ray powder diffraction (*e.g.* Kohl *et al.*, 2001 ▸; Ogienko *et al.*, 2006 ▸) to study materials such as water ice, energetic (explosive) compounds and gas hydrates. In this paper, we describe a high-pressure apparatus for the synthesis of planetary ices in the range 0 ≤ *P* ≤ 2 GPa and 80 ≤ *T* ≤ 310 K, rapid chilling of the samples to 80 K whilst under load, and their recovery into liquid nitro­gen. Sample volumes in excess of 2000 mm^3^ may be prepared in a single loading, sufficient for both X-ray and neutron powder diffraction. We illustrate the use of this apparatus, in combination with our new cold-loadable (PheniX-FL) X-ray stage, with two examples. The first of these is the preparation and study of the high-pressure ice polymorph ice VI, which is thermodynamically stable only above ∼0.6 GPa. The second example is our discovery of a new high-pressure polymorph of MgSO_4_·5H_2_O formed by high-pressure dehydration of epsomite, MgSO_4_·7H_2_O, for which we have determined the crystal structure from its X-ray powder diffraction pattern.

## 2. The high-pressure apparatus   

The design criteria for the apparatus were that it should (i) be capable of achieving high compression ratios, in excess of 30% – as might be found, for example, when water ice is compressed to 2 GPa (*e.g.* Fortes, Wood *et al.*, 2012 ▸); (ii) allow rapid cooling and easy sample recovery into liquid nitro­gen; (iii) enable changes in sample volume to be monitored in real time; and (iv) allow an ultrasonic transducer to be mounted to measure the wave speeds in the sample. These criteria are most easily satisfied by a piston-cylinder cell.

**Figure 1:**
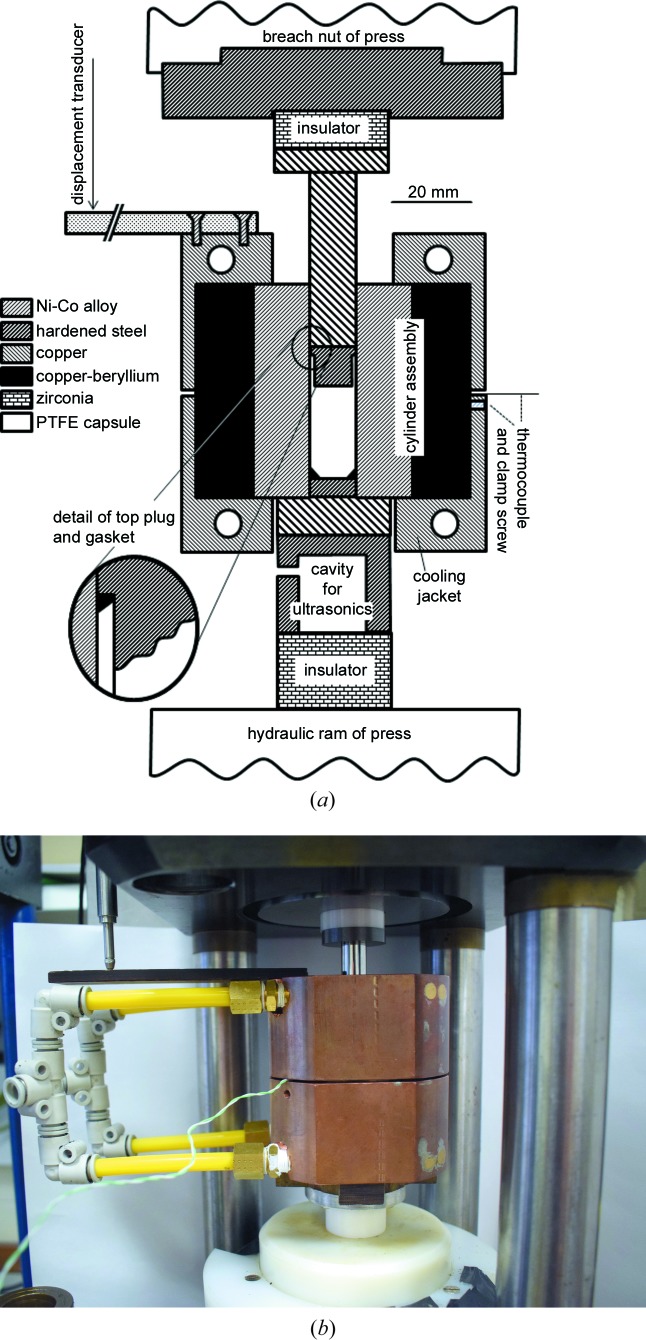
(*a*) A schematic cross section of the piston-cylinder cell for cryo-recovery. (*b*) The piston-cylinder cell, with insulating jackets removed, mounted on a modified Paris–Edinburgh press (one leg of the press has been removed for ease of access). For details see text.

A schematic cross section of our pressure cell is shown in Fig. 1 ▸(*a*). For compactness combined with strength, the cylinder, which has a 12 mm bore, is of the compound fretted type with an inner cylinder (made from nickel–cobalt alloy) held under radial compression by an outer cylinder (made from beryllium–copper alloy). These materials were chosen on the basis of their yield strength and their ductility at low temperatures, allowing safe operation of the cell to pressures of 2 GPa at temperatures down to 80 K. In constructing this unit, the outer surface of the inner cylinder and the inner surface of the outer cylinder are tapered by 1° and the inner cylinder is press-fitted into the heated outer cylinder. The sample is contained within a poly(tetrafluoroethylene) (PTFE) capsule (∼35 mm long) with a 0.5 mm wall thickness. Underneath the bottom end of the sample capsule is a reusable annular gasket made from copper–beryllium alloy (or copper, if working below 1 GPa) and a hardened steel end plug. At the top end of the capsule is another hardened steel plug 10 mm in length (of which 8 mm fits inside the sample capsule) and a smaller annular gasket to prevent extrusion of the PTFE (we have found that no gasket is required if working below 1 GPa). Both of these plugs are relatively loose fits in the bore of the cylinder. The piston, which is 46 mm long, is made from polished tungsten carbide and is a sliding fit in the bore. In our original design, the piston and top plug were made from a single piece of tungsten carbide, but we found that this tended to fracture just above the point where the diameter was reduced to fit inside the sample capsule. At either end of the piston–cylinder cell there are ceramic insulators, made from toughened zirconium oxide, backed with tungsten carbide pressure plates, to isolate the cell thermally from the load frame. Underneath the bottom pressure plate there is a hollow steel cap designed to allow an ultrasonic transducer to be mounted, but we have not yet enabled this facility. Two snugly fitting copper cooling jackets surround the cylinder; the lower of these is clamped onto the cylinder with a nylon-tipped grub screw, which also serves to clamp a thermocouple (currently K-type) onto the body of the cell. The ends of the two copper jackets are drilled to allow nitro­gen from an open-topped Dewar flask to be sucked through them with a pump; this system has the advantage over the use of a pressurized Dewar flask in that the nitro­gen may easily be replenished as often as necessary without any disruption to the cooling. When the cell is in use at low temperatures, these two copper cooling jackets are surrounded by detachable foam-plastic insulation. The thermocouple is not immersed within the sample and so, strictly, it is the temperature of the cell body that is measured, rather than the sample temperature. The thermocouple is, however, mounted at a point far from the flowing refrigerant and so the reading from it reflects the temperature of the whole of the cell body and thus of the sample itself. Similarly, the linear variable displacement transducer (LVDT), which is mounted on the load frame and which bears on a Paxolin plate fastened to the upper cooling jacket, actually measures the change in position of the cell body as load is applied, rather than the change in the length of the sample itself. However, as all of the components of the cell are much less compressible than the samples (the zero-pressure incompressibilities of the samples are typically in the range 10–35 GPa) and as compression is single sided with the cell body resting on the ram of the press, we have found this arrangement to be adequate for recording the changes in volume associated with phase transitions in the sample.

**Figure 2:**
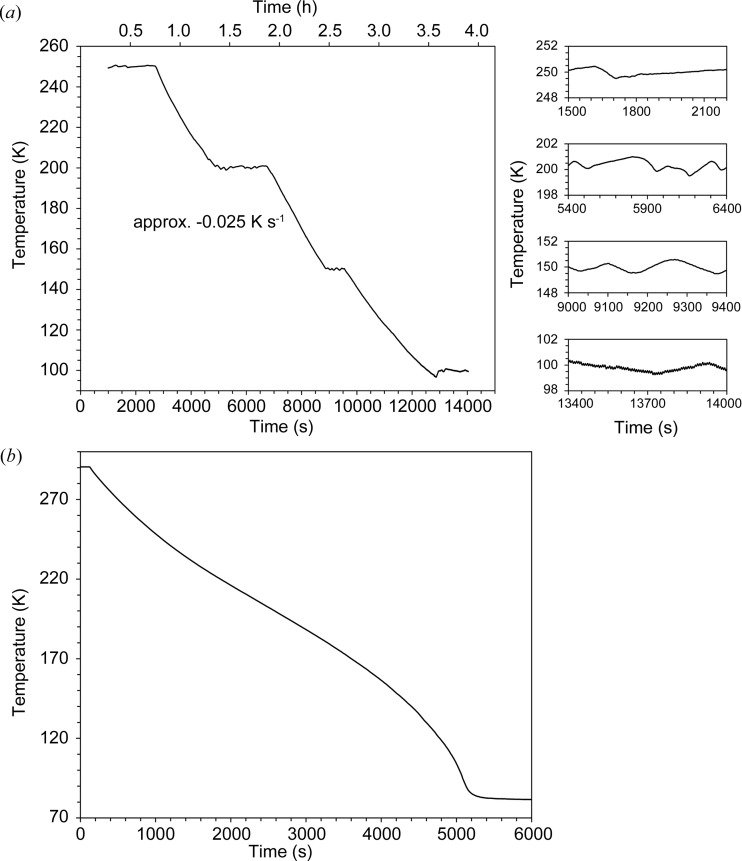
Temperature logs (at 1 s intervals) showing (*a*) the temperature stability achievable by manual control and (*b*) the cooling curve (for a sample of epsomite compressed to ∼2 GPa) when cooling at the maximum rate. For details see text.

The bore of the cell is 113 mm^2^ in area and so (ignoring friction) a load frame of 22.6 tonne capacity is required to reach the maximum design pressure of 2 GPa on the sample. Since it was available, we have used the modified Paris–Edinburgh press described by Dobson *et al.* (2005 ▸) for this purpose (see Fig. 1 ▸ *b*), but this has over ten times the necessary load capacity – any load frame capable of exerting a force of ∼25 tonnes, with a stroke of at least 15 mm, would be suitable. In this Paris–Edinburgh press, a hydraulic oil pressure of 10 bar (1 bar = 10^5^ Pa) equates to a force of 1 tonne on the hydraulic ram. At present, the oil pressure is varied by means of a simple hydraulic hand pump and manual shut-off and bleed valves, with a digital oil-pressure gauge reading to 0.01 bar, but this system could easily be interfaced to an automatic pressure controller if required. Temperature is, at present, also controlled manually by a combination of opening and closing valves on the exhaust side of the pressure cell (so as to admit air from the laboratory as well as nitro­gen gas from the Dewar to the pump), and an in-line heater in the nitro­gen flow on the inlet side. The high thermal mass of the cell means that a temperature stability of ±1 K can be achieved in this way without difficulty (see Fig. 2 ▸ *a*). Fig. 2 ▸(*b*) shows the temperature profile when the cell (containing a sample of MgSO_4_·7D_2_O compressed to ∼2 GPa) is cooled as rapidly as possible; the temperature falls from 290 to 83 K in just under 87 min, giving an average cooling rate of 2.4 K min^−1^. Digital read-outs of the oil pressure in the ram and the dis­place­ment from the LVDT can be continuously logged using the *LabView* software (*e.g.* Elliott *et al.*, 2007 ▸); the temperature is similarly recorded by means of a TC-08 thermocouple logging system from Pico Technology (TC-08 User’s Guide; Pico Technology, 2016 ▸).

For sample recovery, the cell is first cooled under load to 80 K, after which the load is gradually reduced to zero (typically over a period of 5–10 min). The cell is then transferred by means of a swing arm [not shown in Fig. 1 ▸(*b*)] to a low-capacity load frame adjacent to the Paris–Edinburgh press. The tungsten carbide pressure plates and zirconia insulators at the top and bottom of the cell are removed, as is the cap for the ultrasonic transducer. Load is then applied to the piston, and the bottom plug and gasket and the sample capsule (with the top plug and gasket) are pushed out of the cylinder into a plastic beaker filled with liquid nitro­gen. To prevent warming of the sample, the nitro­gen flow through the piston–cylinder cell is maintained throughout this process. For samples which have been compressed to ∼1 GPa we have found sample recovery to be very straightforward. If the cell has been taken to 2 GPa, extrusion of the upper gasket and PTFE capsule has sometimes occurred, making recovery more difficult but still possible; a recent minor modification to the top plug and gasket should eliminate, or greatly reduce, these difficulties. On occasion (possibly as the result of ice forming around the bottom plug) sample recovery can be quite violent, and so we have found it advisable to place the plastic beaker into which the sample is to be recovered (which can shatter) inside a stainless steel container and to employ suitable shielding around the sample-recovery press.

After recovery of the sample into liquid nitro­gen, it is extracted from the capsule and ground under liquid nitro­gen in a stainless steel cryo-mortar. We have found that a sharp 6 mm wood chisel (pre-cooled in liquid nitro­gen) provides an effective tool for opening the capsule. We have also found it very helpful to use capsules made from coloured PTFE, as the majority of our planetary ice samples are white. To avoid contamination of the sample by ice formed by condensation of atmospheric water vapour, the cryo-grinding is best carried out in a cold room (or other dry environment), but this is not essential. If the sample is to be examined by X-ray powder diffraction, it is then loaded into a pre-cooled sample carrier and put into the PheniX-FL low-temperature stage on the diffractometer whilst remaining at a temperature close to 80 K throughout, as described in our accompanying paper (Wood *et al.*, 2018 ▸). Alternatively, if neutron powder diffraction is to be used, a procedure such as that described by Fortes *et al.* (2010 ▸) can be adopted.

## 3. Examples   

We present here two examples of the use of this high-pressure apparatus in combination with the PheniX-FL low-temperature stage for X-ray powder diffraction described in the accompanying paper (Wood *et al.*, 2018 ▸). The first example describes the synthesis of ice VI, while the second describes the use of the apparatus to solve the crystal structure of a new high-pressure polymorph of MgSO_4_·5H_2_O formed by partial dehydration of MgSO_4_·7H_2_O at high pressure.

### 3.1. Ice VI   

**Figure 3:**
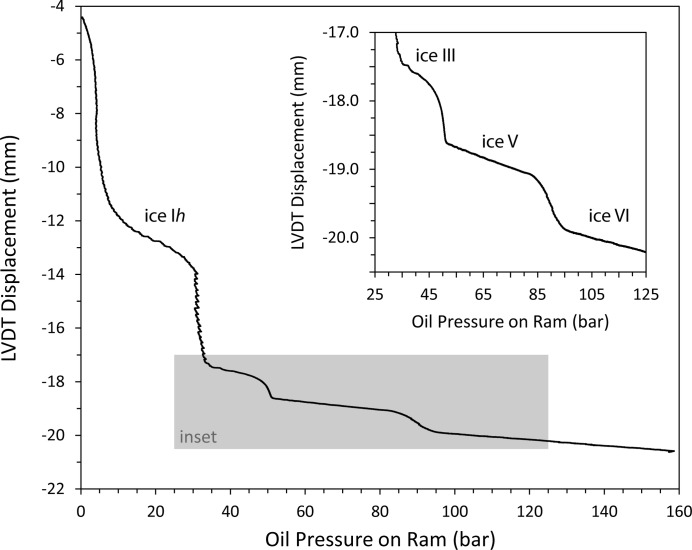
The compression of ice at 250 K. The onsets of the phase transitions between ice I*h*, ice III, ice V and ice VI (at approximately 31, 46 and 84 bar, respectively) are clearly visible. The inset shows the two higher-pressure transitions in more detail.

To prepare the ice VI sample, deionized water was frozen inside a PTFE sample capsule, which was then put into the piston–cylinder cell, pre-cooled to ∼250 K in our cold room. The cell was then mounted on the Paris–Edinburgh press and the temperature was set to 250 ± 1 K. Fig. 3 ▸ shows the LVDT reading plotted against oil pressure in the ram of the Paris–Edinburgh press for compression of this ice at 250 K to a maximum oil pressure of 158 bar, equivalent (ignoring friction) to a pressure on the sample of 1.4 GPa. Along this isotherm, four polymorphs of ice (I*h*, III, V and VI) are expected, with phase boundaries at 0.21, 0.34 and 0.62 GPa (Bridgman, 1912 ▸). It can be seen in Fig. 3 ▸ that, following the initial removal of void space, transitions clearly occur beginning at about 31, 46 and 84 bar, equivalent to sample pressures of 0.27, 0.40 and 0.73 GPa, respectively. These transition pressures do not correspond exactly to the known phase boundaries for two reasons. Firstly, some of the load applied by the press is taken up by overcoming friction; secondly, as remarked by Bridgman (1912 ▸), the transitions can be very sluggish at 250 K and will not proceed rapidly unless overpressure is applied (as our purpose here was mainly just to prepare a sample of ice VI, the entirety of the compression took only about 23 min). The expected volume changes at the three transitions were determined by Bridgman (1912 ▸) to be 5.489, 1.632 and 1.133 Å^3^ per molecule (for I*h*–III, III–V and V–VI, respectively). Again, it can be seen from Fig. 3 ▸ that the magnitudes of the observed volume discontinuities correspond quite well with those expected. To measure the size of these discontinuities relative to the sample volume, both the initial length of the fully dense sample and the LVDT reading at the point at which the sample begins to be elastically loaded must be known. The former can be calculated from the mass of the sample (not recorded in the present case), its density at atmospheric pressure and the temperature of the experiment, and the cross-sectional area of the sample capsule; the LVDT reading at the start of true sample compression can be estimated by back-extrapolation of the ice I*h* compression curve (*e.g.* the region between 14.5 and 26 bar) to zero load. However, even when used in the present rather crude way, the apparatus allows the points at which phase transitions occur in the sample to be readily found. The volume change at the transition between ice V and ice VI is ∼5% (*e.g.* Fortes, Wood *et al.*, 2012 ▸) and it is clear from the inset to Fig. 3 ▸ that changes in sample volume of ∼1% should be clearly resolvable with this apparatus.

Having been compressed to ∼1.4 GPa, the sample was cooled under load to 80 K, after which the load was gradually reduced to zero and the sample pushed out into liquid nitro­gen. It was then ground in a cryo-mortar under liquid nitro­gen, loaded into a PheniX-FL sample carrier (pre-cooled in liquid nitro­gen) and put into the PheniX-FL at 80 K; we judged that the sample temperature remained below 90 K throughout this process.

**Figure 4:**
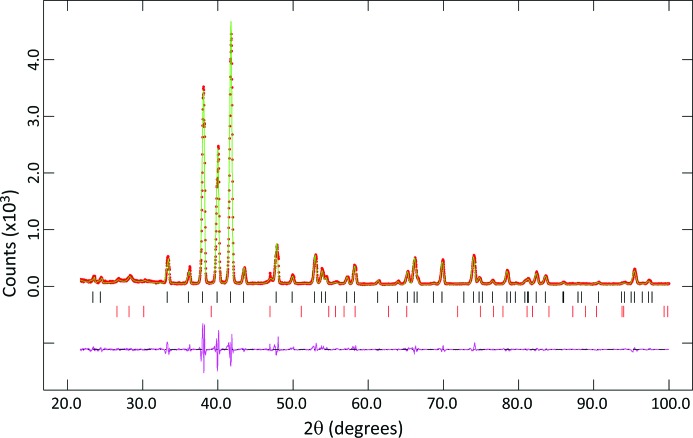
Rietveld refinement of a sample of ice VI at 80 K, prepared by compressing ice to ∼1.4 GPa at 250 K and then recovering the sample into liquid nitro­gen. For details of the data collection see text. The reflection markers are (top down) ice VI and ice I*h* (for further details see text).

Fig. 4 ▸ shows the results of Rietveld refinement of the X-ray powder diffraction pattern collected from this sample at 80 K with our PANalytical X’Pert Pro diffractometer in Bragg–Brentano parafocusing reflection geometry. This diffractometer is equipped with a Ge(111) Johansson geometry focusing monochromator, producing a Co *K*α_1_ incident beam, the wavelength of which was assumed to be 1.788996 Å (Hölzer *et al.*, 1997 ▸). The X-ray tube was operated at 40 kV and 30 mA. Variable-width divergence and anti-scatter slits were used in the incident and diffracted beams, together with a 10 mm wide beam mask in the incident beam, so as to illuminate a constant 10 × 10 mm area of the sample; 0.04 radian Soller slits were present in both the incident and diffracted beams to reduce the axial divergences. The X-ray detector was an X’Celerator position-sensitive detector, covering simultaneously an angular range in 2θ of ±1.061° with an effective fixed step size of 0.0167°. Data were collected for 20 ≤ 2θ ≤ 100° with a data collection time of 130 min.

The intensities of the diffraction pattern were converted from variable- to fixed-divergence slit geometry using software supplied by the manufacturer, and the Rietveld refinement was carried out using the *GSAS* suite of programs (Larson & Von Dreele, 2000 ▸) with the *EXPGUI* graphical interface (Toby, 2001 ▸). It can be seen that a good fit is obtained to the data, on the basis that the sample is composed solely of ice VI with a small amount of contamination by ice I*h* [χ^2^ = 1.875; weighted and unweighted profile *R* factors (including background) 0.1165 and 0.0891, respectively]. The phase proportion of ice I*h* determined from the scale factors is 2.84 (5)% (although the accuracy to which this quantity has been determined is likely to be considerably worse than is indicated by its estimated standard uncertainty; *e.g.* León-Reina *et al.*, 2009 ▸) and the ice I*h* is textured, suggesting that it has formed by frosting of the sample surface.

In the refinement, space group *P*4_2_/*nmc* was assumed for ice VI and the initial atomic coordinates were taken from Kuhs *et al.* (1984 ▸); hydrogen atoms (assumed to be fully disordered) were included in the calculation but their positions were not refined. The unit cell of ice VI contains ten molecules of water; in the setting used by Kuhs *et al.* (1984 ▸) the oxygen atoms occupy the 2*a* positions (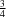, 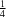, 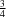) and the 8*g* positions (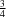, *y*, *z*). Our refined values of the cell parameters were *a* = 6.2527 (8) Å and *c* = 5.7797 (1) Å and the two variable fractional coordinates of the O atom were *y* = 0.5282 (1) and *z* = 0.1284 (2). These cell parameters are in good agreement with those reported at 98 K by Kamb (1965 ▸) by single-crystal X-ray diffraction of ice VI recovered to atmospheric pressure [*a* = 6.27 (1) Å and *c* = 5.79 (1) Å]. Similarly, the fractional coordinates of the oxygen atom in the 8*g* position agree within error with those determined by neutron powder diffraction for deuterated ice VI by Kuhs *et al.* (1984 ▸), who found *y* = 0.5295 (43) and *z* = 0.1339 (38) at 225 K and 1.1 GPa.

Although the agreement between the observed and calculated intensities in Fig. 4 ▸ is very good [a very small correction for preferred orientation, using a spherical-harmonic description (Von Dreele, 1997 ▸), was made for ice VI; texture index = 1.016], it can be seen from the difference profile that the calculated peak shapes correspond less well to those observed, especially for the strong reflections at 2θ ≃ 40°. There are several possible reasons for this: (i) deficiencies in the cryo-grinding and sample loading, leading to crystallites in the sample that are too coarse and a sample surface that is uneven; (ii) microstrain in the sample, which is well outside its stability field; or (iii) ordering of the hydrogen atoms, leading to the formation of triclinic ice XV (Salzman *et al.*, 2009 ▸). The latter possibility can only be properly examined by neutron diffraction, but trial refinements of the data shown in Fig. 4 ▸ using the ice XV unit cell did not appear to produce any significant improvement in the fit.

**Figure 5:**
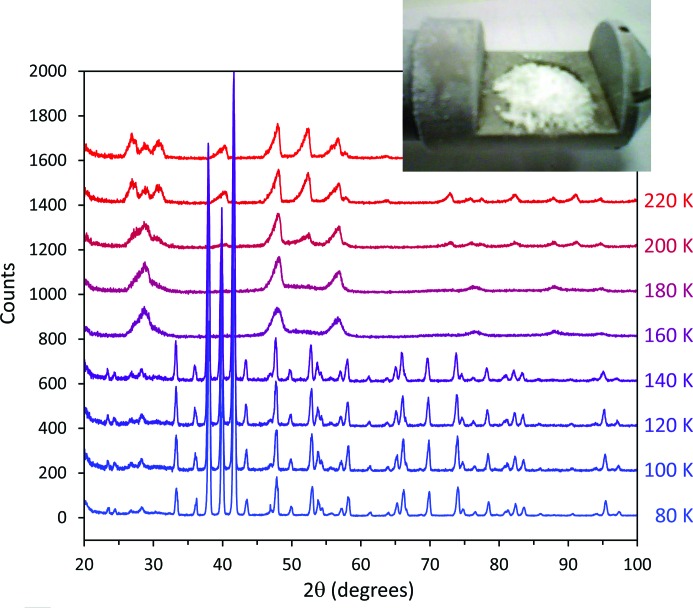
A stack plot of diffraction patterns of ice VI collected on warming the sample. The bottom data set was collected at 80 K; successive scans are at 20 K intervals to 240 K. The heating rate between scans was 2 K min^−1^. The same counting time (39 min) was used for all data collected between 100 and 240 K; the pattern at 80 K (also shown in Fig. 4 ▸), which was counted for 130 min, has been scaled accordingly. For clarity, successive patterns are offset vertically by 200 counts. The breakdown of the sample to a stacking-disordered ‘cubic ice’ by 160 K and its subsequent transformation into poorly crystalline ice I*h* by 220 K can be clearly seen. The inset photograph shows the sample on removal from the diffractometer at 240 K.

Fig. 5 ▸ shows a stack plot of diffraction patterns collected from the ice VI sample in the PheniX-FL at 20 K intervals on warming from 80 to 240 K; the heating rate when changing temperature was 2 K min^−1^. The figure clearly shows the breakdown of the sample, firstly to a stacking-disordered ‘cubic ice’ by 160 K (*cf.* Kuhs *et al.*, 2012 ▸), and subsequently by its transformation into poorly crystalline ice I*h* by 220 K. The inset photograph in the upper right of Fig. 5 ▸ shows the sample on removal from the diffractometer at 240 K. It can be seen that the sample has formed a ‘fluffy mound’, standing proud of the surface of the sample carrier by perhaps as much as 2 mm in the centre, which is perhaps not surprising given the very large volume change effected by the back-transformation from ice VI to ice I*h*. We have observed similar behaviour in the back-transformation by rehydration of samples of MgSO_4_·5H_2_O to MgSO_4_·7H_2_O (see below).

### 3.2. The high-pressure behaviour of epsomite (MgSO_4_·7H_2_O) and the formation of a new high-pressure polymorph of MgSO_4_·5H_2_O   

The experiments on water ice reported above provided us with a means of assessing the capabilities of our new apparatus by applying it to a well known system. In this section, we report new results on the behaviour of synthetic epsomite (MgSO_4_·7H_2_O) at high pressure.

As described by Fortes *et al.* (2017 ▸), magnesium sulfate combines with water to form a wide array of crystalline hydrates; examples are known with the number of water molecules per formula unit *n* = 1, 1.25, 2, 2.5, 3, 4, 5, 6, 7, 9 and 11. The thermodynamically stable solid phase in equilibrium with a saturated aqueous solution of magnesium sulfate at room temperature and pressure is the heptahydrate, which occurs naturally as the mineral epsomite. Investigations of the high-pressure behaviour of epsomite were initiated by Bridgman (1948*a* ▸,*b* ▸), who determined the volumetric compressibility of polycrystalline specimens up to 4 GPa, and the axial incompressibility of single-crystal specimens up to 1 GPa, using a piston–cylinder apparatus. He observed a series of discontinuities in the pressure–volume curves, and identified these as a sluggish phase transition between 1.0 and 1.5 GPa, followed by two further sluggish transitions at ∼2.5 GPa. Subsequently, Livshits *et al.* (1963 ▸) reported a series of phase transitions in the heptahydrate: I → II (∼0.45 GPa), II → III (∼1.2 GPa), III → IV (∼1.6 GPa) and IV → V (∼2.5 GPa).

Our interest in the material more recently is motivated by its likely occurrence as a major rock-forming mineral in extraterrestrial environments, such as the large icy satellites of Jupiter. If hydrated magnesium sulfates were to occur in the mantle of, for example, Ganymede, pressure-induced polymorphism and/or dehydration reactions would produce a layered structure that would have a major influence on mantle convection and heat transport, directly affecting the surface geology and having implications for the presence and long-term stability of subsurface ‘brine’ oceans (*e.g.* Fortes & Choukroun, 2010 ▸; Vance *et al.*, 2014 ▸).

With other colleagues, we carried out a series of ultrasonic and high-pressure neutron powder diffraction experiments on synthetic epsomite (Gromnitskaya *et al.*, 2013 ▸), from which we were able to identify a series of transitions on compression. Between 280 and 295 K, three transitions were observed, occurring at approximately 1.4, 1.6 and 2.5 GPa, in very good agreement with Bridgman’s observations. Between 240 and 280 K there was only a single transition, occurring at a pressure which varied from ∼1.5 GPa at 280 K to ∼2 GPa at 240 K. Below 240 K, no transitions were found. Gromnitskaya *et al.* (2013 ▸) proposed a *P*/*T* diagram in the range 0 < *P* < 2.7 GPa and 230 < *T* < 300 K containing five phase fields, at least some of which were attributed to changes in the magnesium sulfate hydration state associated with incongruent melting, producing a lower hydrate and brine (or a glassy solid). Although well counted neutron powder diffraction data were available for perdeuterated analogues of several of these high-pressure phases, it was not possible to index any of the diffraction patterns unambiguously, and consequently both the crystal structures and hydration states of these materials remained unknown.

In the present work, we have used the combination of our new piston–cylinder cell with cryo-recovery and our PheniX-FL cold stage to prepare the material tentatively labelled ‘Phase III’ by Gromnitskaya *et al.* (2013 ▸) and measure its X-ray powder diffraction pattern with good resolution. It was found that these X-ray data were sufficient to enable us to determine the unit cell and crystal structure of the compound, which proved to be a new high-pressure polymorph of MgSO_4_·5H_2_O, thus revealing at least one of the high-pressure transitions to be a pressure-induced dehydration or incongruent melting.

#### 3.2.1. Sample preparation and data collection   

Synthetic epsomite was obtained by crystallization from a solution of anhydrous magnesium sulfate (ReagentPlus grade ≥99.5%, Sigma–Aldrich) in deionized water. After drying on filter paper the epsomite was ground with an agate pestle and mortar. It was checked by X-ray powder diffraction and found to be phase pure. The sample was then packed into a PTFE capsule, put into the piston–cylinder cell and compressed at room temperature to maximum load (equivalent to ∼2 GPa). As a result of the PTFE capsule splitting, liquid was observed to emerge from the bottom of the cylinder during compression. Although not intentional, it has since become apparent that this splitting of the capsule proved most fortuitous, as it allowed the brine formed on incongruent melting of the epsomite to escape from the system, thereby enabling recovery, after cooling to 80 K, of a single-phase hydrate, without the complication of the presence of high-pressure phases of water ice (see §3.2.5).

As it was known that the transitions at high pressure were sluggish [Gromnitskaya *et al.* (2013 ▸) used compression rates as slow as 10^−3^ GPa min^−1^], the sample was left under maximum load overnight, after which the temperature was reduced to 80 K at an overall cooling rate of 2.4 K min^−1^ and the sample was recovered into liquid nitro­gen. On this occasion, some difficulty was encountered in pushing the sample out of the cylinder and it is possible that the sample may have warmed above 80 K, but it was clear from subsequent analysis that the high-pressure form of the compressed sample had been successfully retained. After cryo-grinding under liquid nitro­gen, the powdered sample was packed into a PheniX-FL sample carrier (also cooled in liquid nitro­gen) and loaded into the PheniX-FL stage on our X-ray powder diffractometer at a temperature of 85 K.

**Figure 6:**
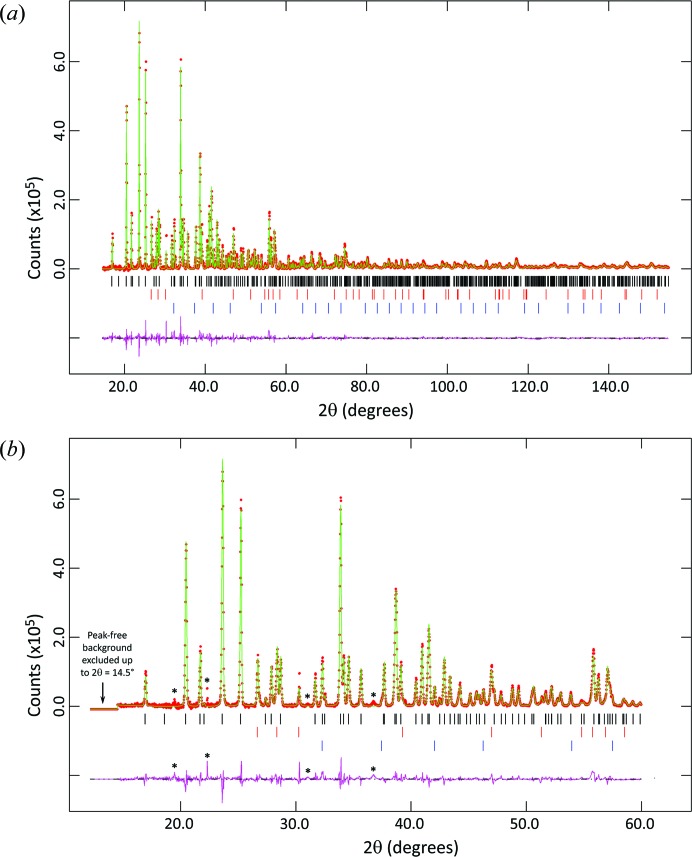
Rietveld refinement of the new MgSO_4_·5H_2_O phase at 85 K. The sample was prepared by compressing MgSO_4_·7H_2_O to ∼2 GPa at ∼293 K and then recovering it into liquid nitro­gen. The upper image (*a*) shows the fit to the complete data set; the lower image(*b*) shows the region 2θ ≤ 60° in more detail. The reflection markers are (bottom up) CO_2_, ice I*h* and MgSO_4_·5H_2_O (for further details see text). The four unindexed reflections marked with an asterisk in panel (*b*) were omitted from the refinement (for details see text).

An initial scan of the diffraction pattern showed that it was different from that of epsomite and so a very well counted data set was collected at 85 K. The configuration of the diffractometer was as described above for ice VI, except that in this case the area of sample illuminated was 10 × 8.5 mm (8.5 mm being the maximum illuminated length available from the divergence of the incident-beam monochromator at 2θ = 155°). The angular range covered was 5 ≤ 2θ ≤ 155°, with a data collection time of 17 h 55 min. These data are shown in Fig. 6 ▸. The advantages of increased resolution in the X-ray data collected outside a high-pressure sample environment are immediately apparent.

Once the data at 85 K had been collected, the behaviour of the material on warming was investigated by making measurements at 20 K intervals from 100 to 260 K, warming the sample at 2 K min^−1^ between each scan. The configuration of the diffractometer was as described above for the data collected at 85 K, except that for these measurements the angular range covered was 5 ≤ 2θ ≤ 60° with a data-collection time of 67.5 min. Finally, a set of five repeated scans (15 ≤ 2θ ≤ 100°, data collection time 131 min for each) was made at 268 K. These data are shown in Figs. 13 and 14.

#### 3.2.2. Structure solution and refinement   

The sharp reflections (FWHM = 0.14° 2θ) in the X-ray powder pattern and the fact that the longest *d* spacings from the crystal were accessible allowed us to index the diffraction pattern. After recognizing the presence of both water ice and solid CO_2_ (the former highly textured and likely to be a surface frosting, the latter a condensed residue from the cryogen used to cool the sample holder), we indexed the Bragg peaks from the unknown high-pressure phase in *DICVOL06* (Boultif & Louër, 2004 ▸). The most favourable solution was orthorhombic with lattice parameters *a* = 12.218 (3), *b* = 11.116 (4), *c* = 5.186 (2) Å and *V* = 704.33 Å^3^, the figures of merit being *M*(14) = 37.2 and *F*(14) = 42.8 (0.0106, 31) (De Wolff, 1968 ▸; Smith & Snyder, 1979 ▸). Since there is a well established linear relationship between the volume per formula unit and the hydration number of MgSO_4_ hydrates (see *e.g.* Fortes, Browning & Wood, 2012*a* ▸,*b* ▸) it was quickly apparent that the unknown phase must be a pentahydrate with *Z* = 4. Analysis of systematic absences indicated a primitive cell with a twofold or 2_1_ axis along **a** and a *b* glide perpendicular to **c**. Four peaks in the diffraction pattern [marked with an asterisk in Fig. 6 ▸(*b*)] could not be indexed on this basis. It was concluded that they arose from a very small amount of an unknown contaminant (or contaminants) in the sample (they did not, for example, correspond to reflections from epsomite) and so they were omitted from the subsequent data analysis. Three of these reflections are broad and very weak; the fourth (at about 22.3° 2θ) has an FWHM of only 0.04°, which is less than the instrumental resolution for normal powder samples at this value of 2θ (about 0.06°, as determined from a silicon standard) and so must presumably have come from a single grain of contaminant.

Attempts were made to solve the structure in a variety of possible space groups belonging to point group *mm*2 using the parallel tempering algorithm in *FOX* (version 1.9.7.1; Favre-Nicolin & Černý, 2002 ▸, 2004 ▸). For the pentahydrate, *FOX* was used to construct ideal MgO_6_ octahedra with Mg—O distances of 2.085 Å, and ideal SO_4_ tetrahedra with S—O distances of 1.495 Å, which were treated as rigid bodies throughout the solution process. Given the possibility that the structure was likely to contain corner- or edge-sharing polyhedra, the calculations were run using the dynamical occupancy correction, which permits *FOX* to merge overlapping atoms – in this instance, co-located oxygen atoms. The crystal structures of H_2_O ice I*h* and solid CO_2_ were explicitly not included since each contributed few overlapping reflections out to a maximum sinθ/λ = 0.3 (where the algorithm is truncated) and could be excluded.

In runs of one million trials each, the crystal structure was optimized against the powder diffraction data. Eventually, in space group *P*2_1_ *nb*, solutions were found that had a low cost function and a high degree of reproducibility and exhibited a sensible arrangement of structural units; these were transformed into the standard setting (space group *Pna*2_1_) and exported for further analysis.

The trial heavy-atom structure was refined by the Rietveld method against the X-ray powder data set using *GSAS*/*EXPGUI*, initially with stiff bond-distance restraints, the Mg—O bond lengths being restrained to 2.09 (5) Å and the S—O bond lengths being restrained to 1.49 (1) Å. Study of the first coordination shell of the water O atoms at the end of this refinement revealed a logical distribution of O⋯O vectors with lengths of 2.7–3.0 Å, consistent with hydrogen-bonded contacts, and so pairs of hydrogen atoms were placed 0.98 Å from each O atom along these vectors.

#### 3.2.3. Location of H atoms   

Since the estimated hydrogen-atom locations as determined above are rather crude, we proceeded to find a more accurate structure using density-functional theory (DFT) calculations with the plane-wave pseudopotential method (Hohenberg & Kohn, 1964 ▸; Kohn & Sham, 1965 ▸). The calculations were carried out using *CASTEP* (Payne *et al.*, 1992 ▸; Segall *et al.*, 2002 ▸; Clark *et al.*, 2005 ▸) in conjunction with the analysis tools in the *Materials Studio* software package (http://accelrys.com). A basis-set cut-off of 1200 eV and a 2 × 5 × 2 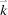-point grid (three irreducible points with ∼0.043 Å^−1^ reciprocal-lattice spacing) were required to achieve convergence of better than 1 × 10^−2^ GPa in the stress and better than 1 × 10^−4^ eV per atom in the total energy. As in our recent work on MgSO_4_·9H_2_O (Fortes *et al.*, 2017 ▸), we used the Wu–Cohen generalized gradient approximation, WC-GGA (Wu & Cohen, 2006 ▸), since this has proven to give highly accurate results with these types of material (see also Tran *et al.*, 2007 ▸; Haas *et al.*, 2009 ▸, 2011 ▸), correcting some of the under-binding commonly encountered in PW91 and PBE simulations. For comparison, a zero-pressure geometry optimization was also done on the structure of the known ambient-pressure 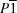 phase of MgSO_4_·5H_2_O (the mineral pentahydrite); convergence criteria were the same as stated above for the *Pna*2_1_ phase, with the exception of using a 4 × 2 × 4 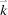-point grid (16 irreducible points with ∼0.045 Å^−1^ reciprocal-lattice spacing).

Structural relaxations under zero-pressure athermal conditions were done using the BFGS method (Pfrommer *et al.*, 1997 ▸). The relaxations were considered to have converged when the forces on each atom were less than 1 × 10^−2^ eV Å^−1^ and each component of the stress tensor was smaller than 0.01 GPa.

As shown in Table 1 ▸, the agreement between the calculated and experimental zero-pressure lattice parameters of both pentahydrate polymorphs is very good. Indeed, given that the unit-cell volume of the Cu analogue of the 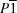 phase (chalcanthite) decreases by 0.14% between 85 and 4.2 K (Schofield & Knight, 2000 ▸), we might anticipate – assuming the volume thermal expansion of the Mg analogue to be similar – that the calculated unit-cell volume of triclinic MgSO_4_·5H_2_O will agree to within ∼0.3% of the true value at 0 K.

The DFT-relaxed zero-pressure athermal structure of *Pna*2_1_ MgSO_4_·5H_2_O including accurate hydrogen-atom positions is provided in CIF format in the supporting information. The refinement of the X-ray powder diffraction data was then completed using the DFT structure as a basis, since the contribution of the hydrogen atoms is not insignificant. Bond-distance restraints for the non-H atoms were turned off and the coordinates of the H atoms were not refined, convergence being achieved with weighted and unweighted profile *R* factors (including background) *wR*_p_ = 0.1116 and *R*_p_ = 0.0806 (χ^2^ = 283.7). The phase proportion of ice I*h* determined from the scale factors is 10.3 (1)% with a high texture index = 2.53; CO_2_ is present with an abundance of 4.1 (1)%. The fit of this model is shown as the green line in Fig. 6 ▸ and included as a CIF in the supporting information. It should be noted that the water ice present in the sample was in the low-pressure ice I*h* phase; this must, therefore, have been introduced either during sample recovery (*e.g.* from frosting on the outside of the piston–cylinder cell or from exsolved brine trapped in the assembly outside the high-pressure volume) or during cryo-grinding of the sample and loading of the PheniX-FL sample carrier.

#### 3.2.4. Description of the structure of the *Pna*2_1_ phase of MgSO_4_·5H_2_O   

**Figure 7:**
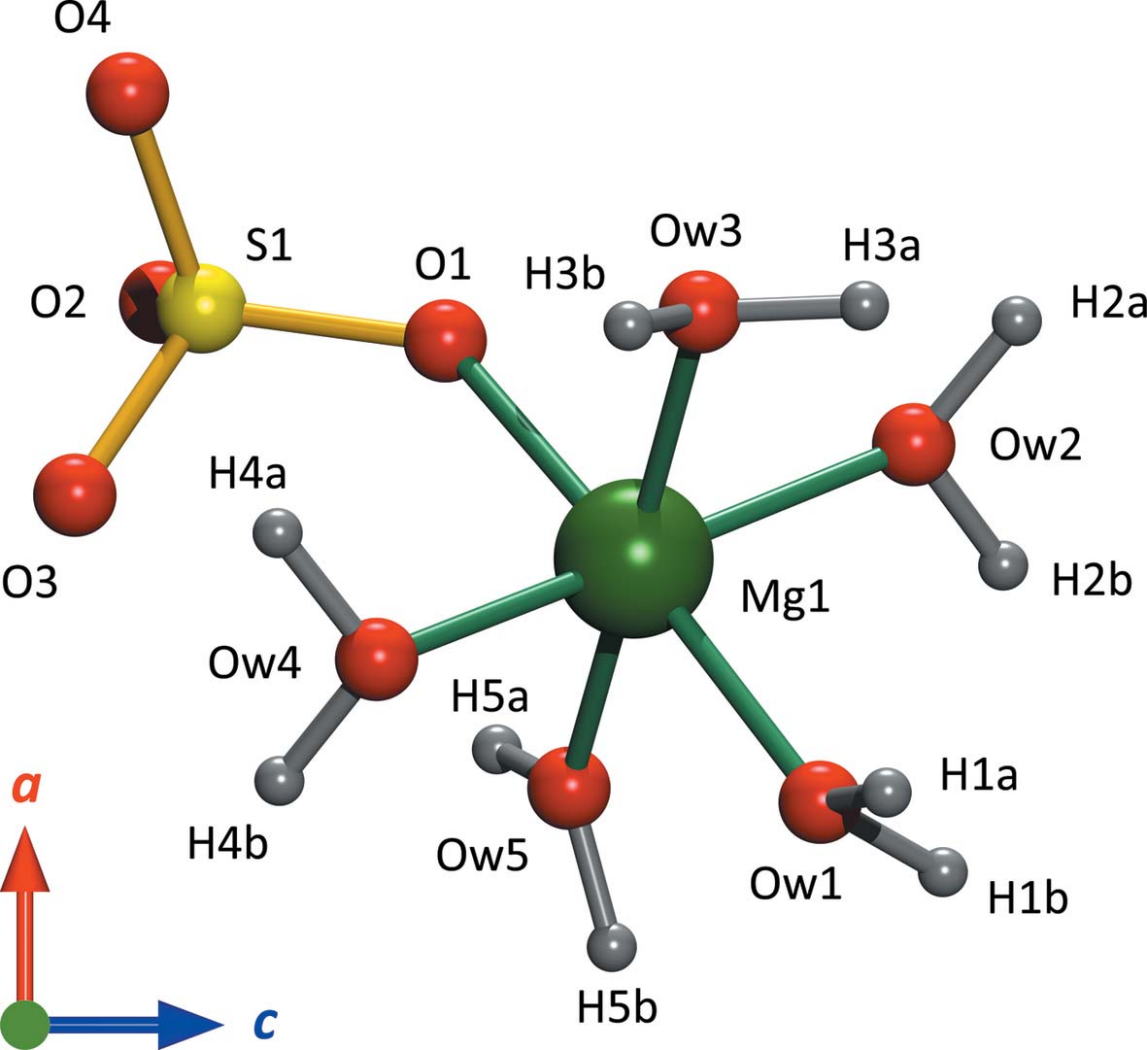
The asymmetric unit of the high-pressure phase of MgSO_4_·5H_2_O, including H atoms derived from *CASTEP* calculations (see text). Graphics produced in *DIAMOND* (Brandenburg & Putz, 2006 ▸) and rendered with *POV-Ray* (Persistence of Vision Team, 2004 ▸).

The structure of this new pentahydrate consists of MgO_6_ and SO_4_ polyhedra arranged into corner-sharing ion pairs of composition Mg(H_2_O)_5_SO_4_ (Fig. 7 ▸); all five water molecules per formula unit are coordinated to the Mg^2+^ ion and there are no interstitial water molecules. This differs from the ambient-pressure 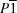 phase of MgSO_4_·5H_2_O (as found in the mineral pentahydrite) described by Baur & Rolin (1972 ▸), in which the sulfate tetrahedra share two corners with adjacent MgO_6_ octahedra, forming infinite corner-linked chains of composition Mg(H_2_O)_4_SO_4_ running along [110]; this leaves the fifth water molecule as an interstitial. Although the pentahydrite structure is very common in related *M* ^2+^-substituted materials, most notably the familiar copper sulfate pentahydrate (chalcanthite), it does not follow the trend seen in other *M* ^2+^SO_4_ hydrates in which the *M* ^2+^ cation becomes completely saturated (*i.e.* achieving a ratio of water to Mg of 6:1) before excess water occurs interstitially. On the other hand, the new MgSO_4_·5H_2_O polymorph does follow the expected trend.

**Figure 8:**
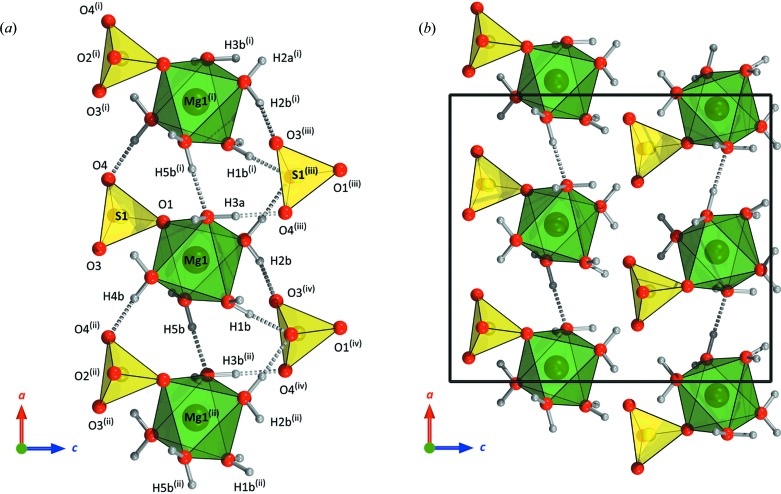
(*a*) A wider view of the structure of the high-pressure phase of MgSO_4_·5H_2_O, showing the hydrogen bonding (dashed rods) between neighbouring Mg(H_2_O)_5_SO_4_ ion pairs. (*b*) A view of the packing in the *ac* plane, emphasizing the hydrogen-bonded chains of MgO_6_ octahedra, linked by O*w*5—H5*b*⋯O*w*3, along the *a* axis. The outline of one unit cell is shown by solid black lines. Symmetry codes: (i) 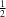
 + *x*, 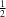
 − *y*, *z*; (ii) *x* − 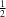, 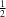
 − *y*, *z*; (iii) 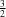
 − *x*, 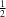
 + *y*, 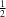
 + *z*; (iv) 1 − *x*, −*y*, 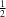
 + *z*.

**Figure 9:**
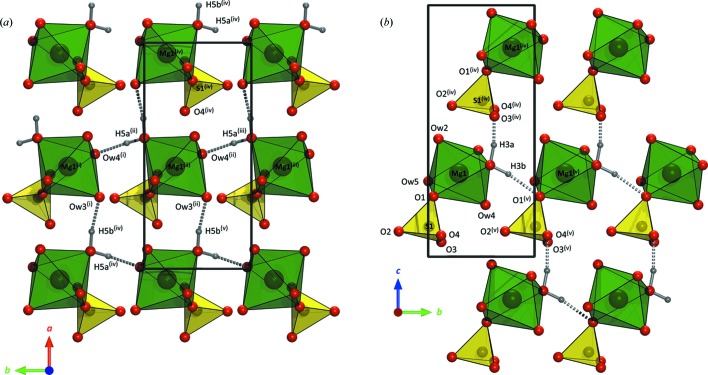
(*a*) The stacking of the polyhedra in the high-pressure phase of MgSO_4_·5H_2_O along the *b* axis, highlighting the hydrogen bonding between adjacent MgO_6_ polyhedra, O*w*5—H5*a*⋯O*w*4 and O*w*5—H5*b*⋯O*w*3. (*b*) A view of the packing down the *a* axis, highlighting the hydrogen bonds donated by O*w*3. The outline of one unit cell is shown by solid black lines. Symmetry codes: (i) 1 − *x*, 2 − *y*, 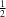
 + *z*; (ii) 1 − *x*, 1 − *y*, 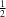
 + *z*; (iii) 1 − *x*, −*y*, 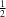
 + *z*; (iv) 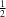
 − *x*, 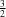
 + *y*, 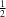
 + *z*; (v) 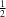
 − *x*, 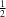
 + *y*, 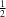
 + *z*; (v) *x*, 1 + *y*, *z*.

The coordination polyhedra in the *Pna2*_1_ phase are comparatively regular (Table 2 ▸). The elongated Mg—O contacts correspond to (i) the oxygen shared with the sulfate anion and (ii) the pair of Mg-coordinated water molecules that accept hydrogen bonds. The local hydrogen bonding of the Mg(H_2_O)_5_SO_4_ ion pairs is illustrated in Fig. 8 ▸(*a*) and the DFT-calculated geometry of these contacts is listed in Table 3 ▸. The lack of interstitial water necessitates direct bridging between adjacent octahedra, O*w*5—H5*b*⋯O*w*3 along the *a* axis (Fig. 8 ▸ *b*) and O*w*5—H5*a*⋯O*w*4 along the *b* axis (Fig. 9 ▸ *a*). Consequently, both O*w*3 and O*w*4 are tetrahedrally coordinated, a relatively common feature in these types of materials across a range of hydration states (see *e.g.* Fortes *et al.*, 2008 ▸). Furthermore, the oxygen atom shared between Mg and S accepts a hydrogen bond (O*w*3—H3*b*⋯O1), the longest of the hydrogen bonds in the structure (Table 3 ▸ and Fig. 9 ▸ *b*). Again, this is not unusual and a similar hydrogen bond donated to a shared oxygen occurs in the low-pressure 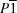 phase of MgSO_4_·5H_2_O (O*w*9—H92⋯O2).

**Figure 10:**
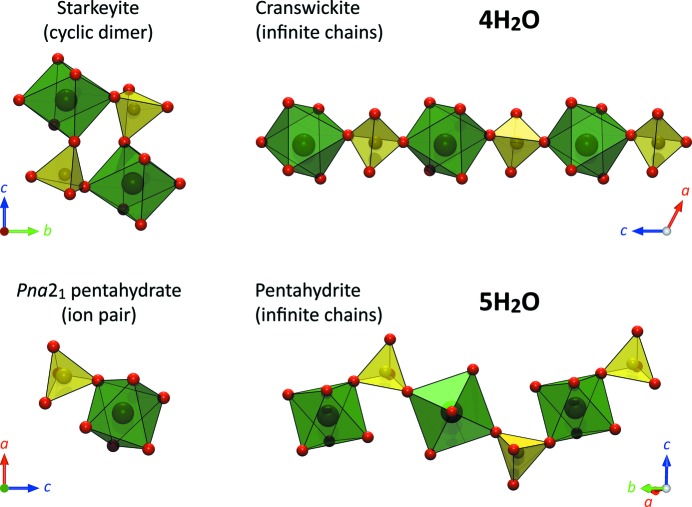
A comparison of the polyhedral connectivity in the two known polymorphs of MgSO_4_·4H_2_O (top) and both the known and newly discovered polymorphs of MgSO_4_·5H_2_O (bottom).

It is worth observing that the tetrahydrate, MgSO_4_·4H_2_O, forms two polymorphs at ambient pressure that exhibit similar structural motifs to the two pentahydrates (Fig. 10 ▸). The recently discovered β form of MgSO_4_·4H_2_O, which occurs naturally as cranswickite (Peterson, 2011 ▸), contains infinite chains of corner-linked MgO_6_ and SO_4_ polyhedra running along the crystal’s *c* axis, similar to (albeit more linear than) the chains occurring in pentahydrite. Similarly, the long-known α form of MgSO_4_·4H_2_O (starkeyite; Baur, 1962 ▸, 1964 ▸) contains isolated cyclic polyhedral units with the composition [Mg(H_2_O)_4_SO_4_]_2_, which is effectively a dimer of the ion pair found in our new high-pressure phase of MgSO_4_·5H_2_O.

#### 3.2.5. Comparison with phases seen *in situ* at high pressure   

**Figure 11:**
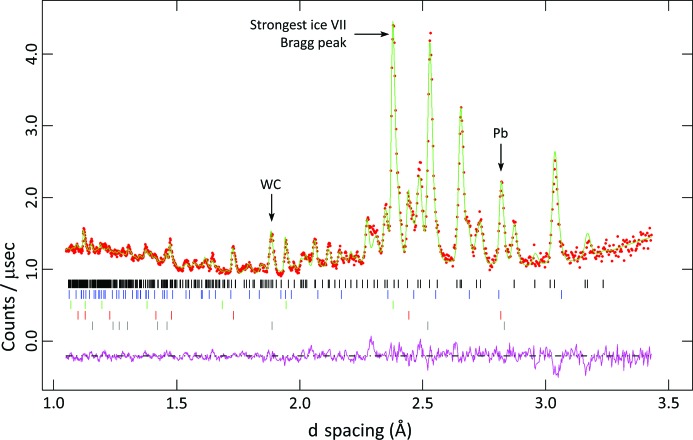
Neutron powder diffraction data and Rietveld refinement of the new MgSO_4_·5D_2_O phase at room temperature under an applied load of 31 tonnes (∼2.17 GPa). These data were collected on the ISIS PEARL diffractometer in 2009 (Gromnitskaya *et al.*, 2013 ▸) but could not be indexed until now. The reflection markers are (top down) MgSO_4_·5D_2_O, ice VI, ice VII, Pb and WC (the strongest Bragg peaks of the various accessory phases are labelled). The refined lattice parameters of MgSO_4_·5D_2_O are *a* = 10.857 (1), *b* = 5.1069 (8), *c* = 12.058 (2) Å and *V* = 668.6 (1) Å^3^.

One of the motivating factors behind this work was to develop a methodology that would enable us to identify the phases observed in a number of high-pressure neutron powder diffraction studies of epsomite. As we reported earlier (Gromnitskaya *et al.*, 2013 ▸), there is a clear and reproducible sequence of changes in the diffraction pattern upon compression of epsomite, none of which we were able to index conclusively. We are now able to confirm that the newly discovered pentahydrate matches one data set collected at ∼2.2 GPa (corresponding to a load of 31 tonnes on the Paris–Edinburgh press) on the PEARL diffractometer at ISIS in 2009 [top of Fig. 1 in the paper by Gromnitskaya *et al.* (2013 ▸)]. The result of a Rietveld refinement of this data set is shown in Fig. 11 ▸; the refinement includes contributions from the anvils (tungsten carbide), Pb (included as a ball of rolled foil to act as a pressure marker), the *Pna*2_1_ phase of MgSO_4_·5D_2_O, and D_2_O ices VI and VII. The weighted and unweighted profile *R* factors (including background) are *wR*_p_ = 0.0339 and *R*_p_ = 0.0370 (χ^2^ = 3.26), respectively. Although a substantial degree of preferred orientation is present in the pentahydrate (texture index = 1.50), this is nevertheless a clear validation of the hydrogen-atom positions, estimated by hand and refined in *CASTEP*.

Considering only the refined scale factors of the ice/hydrate components, the specimen in the high-pressure neutron diffraction experiment consists of 87.1% MgSO_5_·5D_2_O, 3.4% D_2_O ice VI and 9.5% D_2_O ice VII. The calculated phase proportions, assuming they formed from decomposition of deuterated MgSO_4_·7D_2_O, are 84.6% MgSO_5_·5D_2_O and 15.4% D_2_O ice. Hence the observed phase fractions are consistent with the understanding that epsomite has decomposed (whether by exsolution or incongruent melting) to a pentahydrate and water ice.

The interpretation would appear to be that this phase mixture (pentahydrate + ice) is the phase III reported by Gromnitskaya *et al.* (2013 ▸). However, if this were true, we would expect to be able to fit neutron powder diffraction data measured at 23–29 tonnes in the same experiment as the 31 tonnes data set. As shown in Figs. 1 and 10 of the paper by Gromnitskaya *et al.* (2013 ▸) and discussed in the text of that paper, we detected a change in the accessory phase from mainly ice VI to mainly ice VII between 29 and 31 tonnes (commensurate with the pressure of 2.17 GPa obtained from Pb at 31 tonnes); however, the residual hydrate peaks from 22 to 29 tonnes do not match the expected diffraction pattern of the *Pna*2_1_ pentahydrate at all, particularly lacking a strong Bragg peak close to 2.53 Å, despite obvious similarities such as the peak at 3.04 Å.

**Figure 12:**
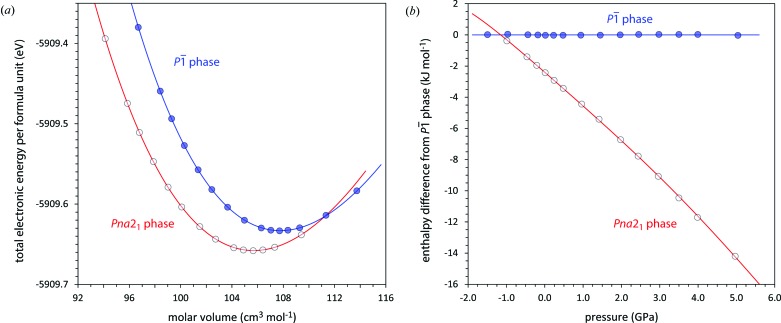
(*a*) The variation in total electronic energy with molar volume, computed in *CASTEP*, for the two MgSO_4_·5H_2_O polymorphs. The solid lines represent Birch–Murnaghan third-order equations of state fitted to the points, the parameters of which are given in Table 4 ▸. (*b*) The variation in enthalpy (in the athermal limit) relative to that of the 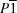 phase.

Hence, in terms of the putative pressure–temperature phase diagram shown in Fig. 12 of the paper by Gromnitskaya *et al.* (2013 ▸), we remain ignorant of the identity of phase II, seen in neutron diffraction data from 20 to 21 tonnes, and of the phase existing between 22 and 29 tonnes. One or both may be additional polymorphs of MgSO_5_·5D_2_O, or else represent an intermediate state between the heptahydrate and penta­hydrate.

#### 3.2.6. Calculated thermodynamic stability   

As a further aid to understanding the stability of the new polymorph with respect to the known ambient-pressure pentahydrate, a series of DFT calculations were done on both phases at a series of fixed pressures from −1.5 to 5.0 GPa. The resulting energy–volume curves are shown in Fig. 12 ▸(*a*) and the parameters obtained by unweighted least-squares fitting to third-order Birch–Murnaghan equations of state are given in Table 4 ▸. The enthalpies of the two phases, normalized by that of the low-pressure phase, are shown in Fig. 12 ▸(*b*); as is also evident from the *E*(*V*) curve, the *Pna*2_1_ phase is thermodynamically more stable (zero kelvin) than the 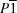 phase at pressures greater than −1.17 GPa. Although the enthalpy difference is not large at 0 GPa (∼2.4 kJ mol^−1^), this grows to ∼14.2 kJ mol^−1^ at 5 GPa.

Further discussion of the DFT calculations is beyond the scope of this paper and these will be reported elsewhere.

#### 3.2.7. Back-transformation/rehydration of MgSO4·5H_2_O   

**Figure 13:**
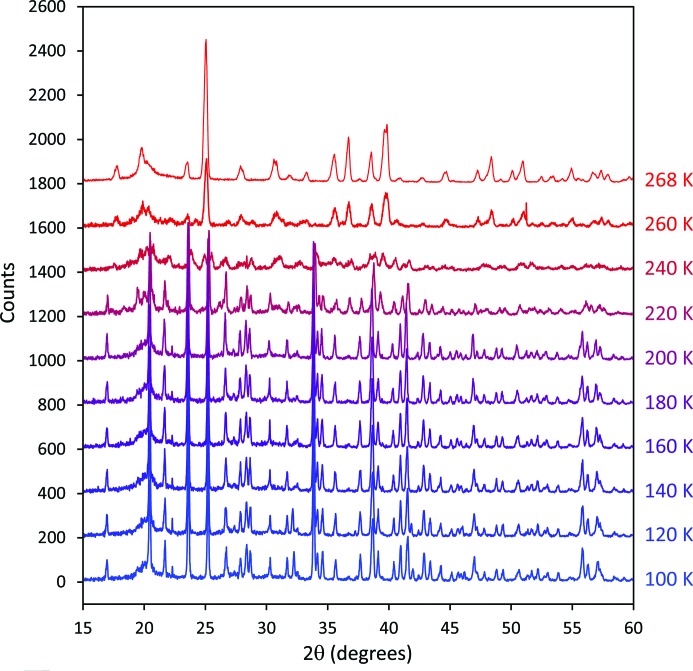
A stack plot of diffraction patterns of the MgSO_4_·5H_2_O sample on warming. The bottom data set was collected at 100 K and successive scans are at 20 K intervals to 260 K, with the uppermost pattern at 268 K; the heating rate between scans was 2 K min^−1^. The same counting time (67.5 min) was used for all data collected between 100 and 260 K; the pattern at 268 K is the summation of five repeated measurements at this temperature with a total counting time of 655 min. For clarity, successive patterns are offset vertically by 200 counts. The rehydration of the sample above 200 K to poorly crystalline MgSO_4_·7H_2_O can be clearly seen.

**Figure 14:**
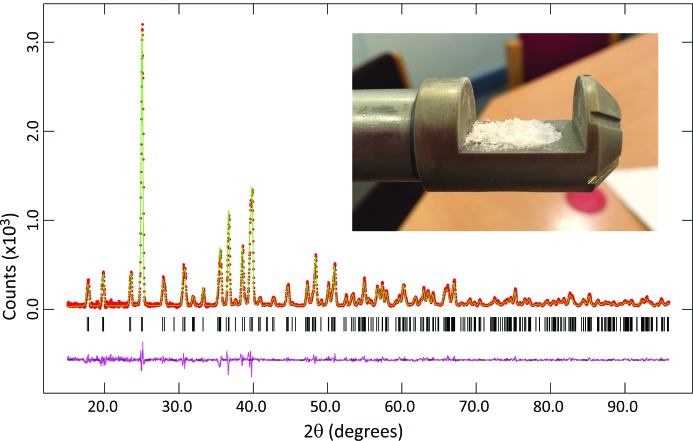
Rietveld refinement of MgSO_4_·7H_2_O formed by rehydration of MgSO_4_·5H_2_O. The data were collected at 268 K and correspond to the uppermost trace in Fig. 13 ▸. The inset photograph shows the sample on removal from the diffractometer at 268 K.

Fig. 13 ▸ shows a stack plot of the X-ray powder diffraction patterns collected (at 20 K intervals) on warming the sample from 100 to 260 K. Little or no change is seen in the data for temperatures up to and including 200 K. At 220 K the diffraction pattern begins to change and by 260 K it resembles strongly the diffraction pattern of epsomite. It would be of some interest to examine the behaviour in the temperature range from 200 to 260 K in more detail, but this is beyond the scope of the present paper. Five repeated measurements were then made at 268 K over a total period of 11 h. No changes were observed across this series and so the data were summed, background stripped in *Origin Pro* (OriginLab, Northampton, Massachusetts, USA) and converted to *GSAS* format for analysis (Fig. 14 ▸). Since the purpose of the subsequent Rietveld refinement of these data was to demonstrate that the back-transformation of the sample had resulted in the formation of crystals of epsomite, we are confident that the adopted procedure is sufficient and no detailed background modelling is required. On recovery of the sample from the diffractometer at 268 K, it was found to have made a small mound in the sample carrier (see inset to Fig. 14 ▸), as had been observed previously for ice VI. Again, this is not surprising given that the volume per MgSO_4_ unit is ∼35% larger in MgSO_4_·7H_2_O than in MgSO_4_·5H_2_O. The Bragg reflections in the diffraction pattern at 268 K are very much broader than would normally be expected from a sample of epsomite measured at room temperature; for example, in the range 18 ≤ 2θ ≤ 31° the epsomite sample prior to compression gave sets of closely spaced Bragg peaks with FWHM values in the range 0.05–0.07° 2θ, whereas in the data shown in Fig. 14 ▸ these doublets are unresolved with combined FWHM values in the range 0.30–0.39° 2θ. However, at least some of the increased width of the Bragg reflections may simply reflect the fact that the sample was in the form of a very loosely packed mound, rather than a well packed powder with a flat surface as is required for Bragg–Brentano geometry.

The fit by Rietveld refinement of the epsomite structure to the combined data set at 268 K is shown in Fig. 14 ▸. The structural model was derived from Fortes *et al.* (2006 ▸) and fitted with no texture correction. Atomic coordinates of the non-H atoms were allowed to vary, subject to moderately stiff bond-distance restraints (Mg—O = 2.07 ± 0.03 Å and S—0 = 1.48 ± 0.01 Å). Within the limits of the data set, the sample appears to consist of pure synthetic epsomite, with no indications from the difference pattern that any additional phases are present. The final weighted and unweighted profile *R* factors (including background) were *wR*_p_ = 0.1233 and *R*_p_ = 0.0822 (χ^2^ = 1.766), respectively. Despite the poorly resolved Bragg reflections in the X-ray pattern, the unit-cell parameters at 268 K [*a* = 11.8687 (4), *b* = 11.9840 (3), *c* = 6.8437 (2) Å and *V* = 973.40 (4) Å^3^] compare extremely well to those obtained at 270 K from MgSO_4_·7D_2_O by Fortes *et al.* (2006 ▸) using high-resolution neutron powder diffraction [*a* = 11.8628 (1), *b* = 11.9877 (1), *c* = 6.8448 (1) Å and *V* = 973.38 (2) Å^3^].

## 4. Concluding remarks   

Using the high-pressure apparatus described here, in combination with the ‘cold-loadable’ PheniX-FL low-temperature stage for X-ray diffraction described in the accompanying paper (Wood *et al.*, 2018 ▸), we have been able to determine definitively that MgSO_4_·7H_2_O undergoes pressure-induced dehydration. It thus conforms to the wider picture of this phenomenon in closely related salt hydrates and more distantly related ices and clathrates. For example, there are precedents for the detection of pressure-induced changes in hydration state in ZnSO_4_·7H_2_O (importantly, resulting in brine being expelled from the bottom of the pressure vessel, as we observed) and in MgSO_4_·6H_2_O (Sood & Stager, 1966 ▸; Churagulov & Kalashnikov, 1969 ▸; Yakuschenko & Churagulov, 1984 ▸; Churagulov, 1987 ▸). We have also recently confirmed that MgSO_4_·11H_2_O (meridianiite) undergoes a pressure-induced dehydration to a previously unknown crystal, MgSO_4_·9H_2_O (Fortes *et al.*, 2017 ▸; Fortes, Fernadez-Alonso *et al.*, 2017 ▸).

Elsewhere, it has been established that ammonia dihydrate and ammonia monohydrate both lose water at high pressure to form the more ammonia-rich compound, ammonia hemihydrate (Wilson *et al.*, 2012 ▸). Equally, there is an extensive literature on the loss of water from the framework of clathrate hydrates; for example methane hydrate increases in CH_4_ concentration across a series of structural phase changes between 0 and 2 GPa, as the material changes from approximately a hexahydrate to a dihydrate (*e.g.* Loveday *et al.*, 2001 ▸, 2003 ▸).

The tendency towards loss of bound water (and concomitant concentration of water in either the liquid or a separate ice phase) at comparatively low pressures is one which deserves wider recognition and application to problems in modelling the internal structure and dynamics of icy planetary bodies. Our new piston–cylinder cell and equipment for the analysis of recovered materials under cryogenic conditions provides an important tool for helping to investigate these important materials.

## Supplementary Material

Crystal structure: contains datablock(s) XU2692_BG_H_publ, XU2692_BG_H_overall, XU2692_BG_H_phase_1, XU2692_BG_H_phase_2, XU2692_BG_H_phase_3, XU2692_BG_H_p_01. DOI: 10.1107/S1600576718003977/ks5581sup1.cif

CIF for ab initio simulation. DOI: 10.1107/S1600576718003977/ks5581sup2.txt

## Figures and Tables

**Table 1:** DFT-calculated zero-pressure lattice parameters compared with the available experimental data

	Low-pressure 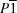			High-pressure *Pna*2_1_		
	Experimental†	Calculated	Difference (%)	Experimental‡	Calculated	Difference (%)
*a* (Å)	6.314 (5)	6.164767	−2.4	11.1252 (1)	10.97749	−1.3
*b* (Å)	10.505 (18)	10.575703	0.7	5.1869 (1)	5.19987	0.3
*c* (Å)	6.030 (6)	6.036037	0.1	12.2180 (1)	12.29529	0.6
α (°)	81.1 (2)	81.76228	0.8	90	90	
β (°)	109.8 (2)	109.75219	−0.0	90	90	
γ (°)	105.08 (5)	104.48120	−0.6	90	90	
*V* (Å^3^)	362.4 (9)	357.8623	−1.3	705.05 (1)	701.8339	−0.5

† Room-temperature single-crystal X-ray diffraction data (Baur & Rolin, 1972 ▸).

‡ 85 K X-ray powder diffraction data, this work.

**Table 2:** DFT-calculated geometry of the coordination polyhedra in the *Pna*2_1_ phase of MgSO_4_·5H_2_O Superscripts after the sulfate oxygens denote the number of hydrogen bonds accepted by each. The superscript dagger symbol (†) after selected Mg-coordinated oxygens denotes those that accept a hydrogen bond (*cf.* Table 3).

S—O1^(1)^	1.4901 Å	O1—S—O2	107.760°
S—O2^(3)^	1.5071 Å	O1—S—O3	110.485°
S—O3^(2)^	1.4800 Å	O1—S—O4	110.623°
S—O4^(2)^	1.4836 Å	O2—S—O3	109.243°
Volume SO_4_	1.6976 Å^3^	O2—S—O4	108.365°
Distribution index	0.00567	O3—S—O4	110.293°
Bond-angle variance	1.4466°^2^		
			
Mg—O1^†^	2.1305 Å	O1—Mg—O*w*1	170.908°
Mg—O*w*1	2.0693 Å	O1—Mg—O*w*2	86.934°
Mg—O*w*2	2.0746 Å	O1—Mg—O*w*3	94.996°
Mg—O*w*3^†^	2.1624 Å	O1—Mg—O*w*4	98.412°
Mg—O*w*4^†^	2.1065 Å	O1—Mg—O*w*5	83.638°
Mg—O*w*5	2.0686 Å	O*w*1—Mg—O*w*2	90.807°
Volume MgO_6_	12.2543 Å^3^	O*w*1—Mg—O*w*3	93.720°
Distribution index	0.01483	O*w*1—Mg—O*w*4	85.038°
Quadratic elongation	1.0073	O*w*1—Mg—O*w*5	87.617°
Bond-angle variance	24.9807°^2^	O*w*2—Mg—O*w*3	87.898°
		O*w*2—Mg—O*w*4	170.778°
		O*w*2—Mg—O*w*5	91.405°
		O*w*3—Mg—O*w*4	84.169°
		O*w*3—Mg—O*w*5	178.500°
		O*w*4—Mg—O*w*5	96.633°

**Table 3:** DFT-calculated geometry (Å, °) of the hydrogen bonds in the *Pna*2_1_ phase of MgSO_4_·5H_2_O

	O—H	H—O—H	H⋯O	O⋯O	O—H⋯O
O*w*1—H1*a*⋯O3^i^	0.9876	101.379	1.7550	2.6922	157.084
O*w*1—H1*b*⋯O2^ii^	0.9918		1.6884	2.6603	165.508
O*w*2—H2*a*⋯O2^iii^	0.9856	106.856	1.8368	2.8181	173.396
O*w*2—H2*b*⋯O3^ii^	0.9941		1.6762	2.6543	167.019
O*w*3—H3*a*⋯O4^iii^	1.0026	105.227	1.7278	2.7202	169.737
O*w*3—H3*b*⋯O1^iv^	0.9909		1.9012	2.8826	170.153
O*w*4—H4*a*⋯O2^iv^	0.9983	107.472	1.7063	2.6857	165.966
O*w*4—H4*b*⋯O4^v^	1.0022		1.7051	2.7029	173.231
O*w*5—H5*a*⋯O*w*4^vi^	0.9920	106.839	1.7914	2.7816	175.757
O*w*5—H5*b*⋯O*w*3^v^	0.9899		1.7957	2.7773	170.753

Symmetry codes: (i) 1 − *x*, 1 − *y*, 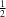
 + *z*; (ii) 1 − *x*, −*y*, 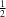
 + *z*; (iii) 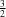
 − *x*, 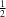
 + *y*, 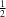
 + *z*; (iv) *x*, 1 + *y*, *z*; (v) *x* − 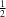, 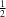
 − *y*, *z*; (vi) *x*, *y* − 1, *z*.

**Table 4:** Third-order Birch–Murnaghan equation of state parameters obtained by fitting to the energy–volume curves of low-pressure pentahydrite and the new high-pressure polymorph calculated with *CASTEP*

	Low-pressure 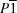	High-pressure *Pna*2_1_
*V*_0_ (cm^3^ mol^−1^)	107.772 (9)	105.696 (10)
*K*_0_ (GPa)	33.0 (1)	31.4 (2)
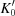	6.4 (1)	5.4 (1)
*E*_0_ (eV per formula unit)	−5909.6330 (1)	−5909.6578 (1)

## References

[bb1] Baur, W. H. (1962). *Acta Cryst.* **15**, 815–826.

[bb2] Baur, W. H. (1964). *Acta Cryst.* **17**, 863–869.

[bb3] Baur, W. H. & Rolin, J. L. (1972). *Acta Cryst.* B**28**, 1448–1455.

[bb4] Boultif, A. & Louër, D. (2004). *J. Appl. Cryst.* **37**, 724–731.

[bb52] Brandenburg, K. & Putz, H. (2006). *DIAMOND*. Crystal Impact GbR, Bonn, Germany. http://www.crystalimpact.com/diamond.

[bb5] Bridgman, P. W. (1912). *Proc. Am. Acad. Arts Sci.* **47**, 441–558.

[bb6] Bridgman, P. W. (1948*a*). *Proc. Am. Acad. Arts Sci.* **76**, 71–87.

[bb7] Bridgman, P. W. (1948*b*). *Proc. Am. Acad. Arts Sci.* **76**, 89–99.

[bb8] Churagulov, B. R. (1987). *Zh. Neorg. Khim.* **32**, 2527–2536.

[bb9] Churagulov, B. R. & Kalashnikov, Ya. A. (1969). *Russ. J. Phys. Chem.* **43**, 258–262.

[bb10] Clark, S. J., Segall, M. D., Pickard, C. J., Hasnip, P. J., Probert, M. I. J., Refson, K. & Payne, M. C. (2005). *Z. Kristallogr.* **220**, 567–570.

[bb11] De Wolff, P. M. (1968). *J. Appl. Cryst.* **5**, 108–113.

[bb12] Dobson, D. P., Mecklenburgh, J., Alfè, D., Wood, I. G. & Daymond, M. R. (2005). *High. Pressure Res.* **25**, 107–118.

[bb13] Elliott, C., Vijayakumar, V., Zink, W. & Hansen, R. (2007). *J. Assoc. Lab. Autom.* **12**, 17–24.

[bb14] Favre-Nicolin, V. & Černý, R. (2002). *J. Appl. Cryst.* **35**, 734–743.

[bb15] Favre-Nicolin, V. & Černý, R. (2004). *Z. Kristallogr.* **219**, 847–856.

[bb16] Fortes, A. D., Browning, F. & Wood, I. G. (2012*a*). *Phys. Chem. Miner.* **39**, 419–441.

[bb17] Fortes, A. D., Browning, F. & Wood, I. G. (2012*b*). *Phys. Chem. Miner.* **39**, 443–454.

[bb18] Fortes, A. D. & Choukroun, M. (2010). *Space Sci. Rev.* **153**, 185–218.

[bb19] Fortes, A. D., Fernandez-Alonso, F., Tucker, M. & Wood, I. G. (2017). *Acta Cryst.* B**73**, 33–46.

[bb20] Fortes, A. D., Knight, K. S. & Wood, I. G. (2017). *Acta Cryst.* B**73**, 47–64.

[bb21] Fortes, A. D., Wood, I. G., Alfredsson, M., Vočadlo, L. & Knight, K. S. (2006). *Eur. J. Mineral.* **18**, 449–462.

[bb22] Fortes, A. D., Wood, I. G. & Knight, K. S. (2008). *Phys. Chem. Miner.* **35**, 207–221.

[bb23] Fortes, A. D., Wood, I. G. & Knight, K. S. (2010). *J. Appl. Cryst.* **43**, 328–336.

[bb24] Fortes, A. D., Wood, I. G., Tucker, M. G. & Marshall, W. G. (2012). *J. Appl. Cryst.* **45**, 523–534.

[bb25] Gromnitskaya, E. L., Yagafarov, O. F., Lyapin, A. G., Brazhkin, V. V., Wood, I. G., Tucker, M. G. & Fortes, A. D. (2013). *Phys. Chem. Miner.* **40**, 271–285.

[bb26] Haas, P., Tran, F. & Blaha, P. (2009). *Phys. Rev. B*, **79**, 085104.

[bb27] Haas, P., Tran, F., Blaha, P. & Schwarz, K. (2011). *Phys. Rev. B*, **83**, 205117.

[bb28] Hansen, T. C., Koza, M. M., Lindner, P. & Kuhs, W. F. (2008). *J. Phys. Condens. Matter*, **20**, 285105.

[bb29] Hohenberg, P. & Kohn, W. (1964). *Phys. Rev.* **136**, B864–B871.

[bb30] Hölzer, G., Fritsch, M., Deutsch, M., Härtwig, J. & Förster, E. (1997). *Phys. Rev. A*, **56**, 4554–4568.

[bb32] Kamb, B. (1965). *Science*, **150**, 205–209. 10.1126/science.150.3693.205 17787274

[bb33] Klotz, S., Besson, J. M., Hamel, G., Nelmes, R. J., Loveday, J. S. & Marshall, W. G. (1999). *Nature*, **398**, 681–684.

[bb34] Klotz, S., Strässle, Th., Salzmann, C. G., Philippe, J. & Parker, S. F. (2005). *Europhys. Lett.* **72**, 576–582.

[bb35] Kohl, I., Mayer, E. & Hallbrucker, A. (2001). *Phys. Chem. Chem. Phys.* **3**, 602–605.

[bb36] Kohn, W. & Sham, L. J. (1965). *Phys. Rev.* **140**, A1133–A1138.

[bb38] Kuhs, W. F., Finney, J. L., Vettier, C. & Bliss, D. V. (1984). *J. Chem. Phys.* **81**, 3612–3623.

[bb39] Kuhs, W. F., Sippel, C., Falenty, A. & Hansen, T. C. (2012). *Proc. Natl Acad. Sci. USA*, **109**, 21259–21264. 10.1073/pnas.1210331110 PMC3535660 23236184

[bb40] La Placa, S. J., Hamilton, W. C., Kamb, B. & Prakash, A. (1973). *J. Chem. Phys.* **58**, 567–580.

[bb41] Larson, A. C. & Von Dreele, R. B. (2000). *GSAS*. Report LAUR 86-748. Los Alamos National Laboratory, New Mexico, USA. http://www.ncnr.nist.gov/xtal/software/gsas.html.

[bb42] León-Reina, L., De la Torre, A. G., Porras-Vázquez, J. M., Cruz, M., Ordonez, L. M., Alcobé, X., Gispert-Guirado, F., Larrañaga-Varga, A., Paul, M., Fuellmann, T., Schmidt, R. & Aranda, M. A. G. (2009). *J. Appl. Cryst.* **42**, 906–916.

[bb43] Livshits, L. D., Genshaft, Yu. S. & Ryabin, Yu. N. (1963). *Russ. J. Inorg. Chem.* **8**, 676–678.

[bb44] Loveday, J. S., Nelmes, R. J., Guthrie, M., Klug, D. D. & Tse, J. S. (2001). *Phys. Rev. Lett.* **87**, 215501. 10.1103/PhysRevLett.87.215501 11736347

[bb45] Loveday, J. S., Nelmes, R. J., Klug, D. D., Tse, J. S. & Desgreniers, S. (2003). *Can. J. Phys.* **81**, 539–544.

[bb46] Millar, D. I. A., Oswald, I. D. H., Barry, C., Francis, D. J., Marshall, W. G., Pulham, C. R. & Cumming, A. S. (2010). *Chem. Commun.* **46**, 5662–5664. 10.1039/c0cc00368a 20617273

[bb47] Ogienko, A. G., Kurnosov, A. V., Manakov, A. Y., Larionov, E. G., Ancharov, A. I., Sheromov, M. A. & Nesterov, A. N. (2006). *J. Phys. Chem. B*, **110**, 2840–2846. 10.1021/jp053915e 16471893

[bb48] Payne, M. C., Teter, M. P., Allan, D. C., Arias, T. A. & Joannopoulos, J. D. (1992). *Rev. Mod. Phys.* **64**, 1045–1097.

[bb51] Persistence of Vision Team (2004). *POV-Ray – Persistence of Vision Raytracer*. Version 3.6. Persistence of Vision Pty. Ltd, Victoria, Australia. http://www.povray.org/.

[bb49] Peterson, R. C. (2011). *Am. Mineral.* **96**, 869–877.

[bb50] Pfrommer, B. G., Côté, M., Louie, S. G. & Cohen, M. L. (1997). *J. Comput. Phys.* **131**, 233–240.

[bb53] Salzmann, C. G., Radaelli, P. G., Mayer, E. & Finney, J. L. (2009). *Phys. Rev. Lett.* **103**, 105701. 10.1103/PhysRevLett.103.105701 19792330

[bb54] Schofield, P. F. & Knight, K. S. (2000). *Physica B*, **276–278**, 897–898.

[bb55] Segall, M. D., Lindan, P. J. D., Probert, M. J., Pickard, C. J., Hasnip, P. J., Clark, S. J. & Payne, M. C. (2002). *J. Phys. Condens. Matter*, **14**, 2717–2744.

[bb56] Smith, G. S. & Snyder, R. L. (1979). *J. Appl. Cryst.* **12**, 60–65.

[bb57] Sood, R. R. & Stager, R. A. (1966). *Science*, **154**, 388–390. 10.1126/science.154.3747.388 17751707

[bb58] Pico Technology Limited (2016). *USB TC-08 Thermocouple Logger User’s Guide*. Report usbtc08.en r8, 2016-04-04. https://www.picotech.com/download/manuals/USBTC08UsersGuide.pdf.

[bb59] Toby, B. H. (2001). *J. Appl. Cryst.* **34**, 210–213.

[bb60] Tran, F., Laskowski, R., Blaha, P. & Schwarz, K. (2007). *Phys. Rev. B*, **75**, 115131.

[bb61] Vance, S., Bouffard, M., Choukroun, M. & Sotin, C. (2014). *Planet. Space Sci.* **96**, 62–70.

[bb62] Von Dreele, R. B. (1997). *J. Appl. Cryst.* **30**, 517–525.

[bb63] Wilson, C. W., Bull, C. L., Stinton, G. & Loveday, J. S. (2012). *J. Chem. Phys.* **136**, 094506. 10.1063/1.3686870 22401451

[bb64] Wood, I. G., Fortes, A. D., Dobson, D. P., Wang, W., Pajdzik, L. & Cosier, J. (2018). *J. Appl. Cryst.* **51**, 685–691. 10.1107/S1600576718003965 PMC5988005 29896057

[bb65] Wu, Z. & Cohen, R. E. (2006). *Phys. Rev. B*, **73**, 235116.

[bb66] Yakuschenko, A. N. & Churagulov, B. R. (1984). *Zh. Fiz. Khim.* **58**, 311–314.

